# Interrater and Intrarater Reliability of Cranial Anthropometric Measurements in Infants with Positional Plagiocephaly

**DOI:** 10.3390/children7120306

**Published:** 2020-12-17

**Authors:** Iñaki Pastor-Pons, María Orosia Lucha-López, Marta Barrau-Lalmolda, Iñaki Rodes-Pastor, Ángel Luis Rodríguez-Fernández, César Hidalgo-García, Jose Miguel Tricás-Moreno

**Affiliations:** 1 Instituto de Terapias Integrativas, 50001 Zaragoza, Spain; inakipas@gmail.com (I.P.-P.); martabarraulalmolda@gmail.com (M.B.-L.); ikirodes95@gmail.com (I.R.-P.); 2 Departamento de Fisiatría y Enfermería, Unidad de Investigación en Fisioterapia, Facultad de Ciencias de la Salud, Universidad de Zaragoza, 50009 Zaragoza, Spain; hidalgo@unizar.es (C.H.-G.); jmtricas@unizar.es (J.M.T.-M.); 3 Departamento de Fisioterapia, Facultad de Medicina, Universidad San Pablo CEU, 28925 Alcorcón, Spain; alrodfer@ceu.es

**Keywords:** positional plagiocephaly, deformational plagiocephaly, brachycephaly, head shape, anthropometry, head circumference

## Abstract

(1) Background: anthropometric measurements with calipers are used to objectify cranial asymmetry in positional plagiocephaly but there is controversy regarding the reliability of different methodologies. Purpose: to analyze the interrater and intrarater reliability of direct anthropometric measurements with caliper on defined craniofacial references in infants with positional plagiocephaly. (2) Methods: 62 subjects (<28 weeks), with a difference of at least 5 mm between cranial diagonal diameters. Maximal cranial circumference, length and width and diagonal cranial diameters were measured. Intrarater (2 measurements) and interrater (2 raters) reliability was analyzed. (3) Results: intra- and interrater reliability of the maximal cranial length and width and right cranial diagonal was excellent: intraclass correlation coefficient (ICC) > 0.9. Intrarater and interrater reliability for the left cranial diagonal was excellent: ICC > 0.9 and difference in agreement in the Bland-Altman plot 0.0 mm, respectively. Intrarater and interrater reliability for the maximal cranial circumference was good: differences in agreement in Bland-Altman plots: intra: −0.03 cm; inter: −0.12 cm. (4) Conclusions: anthropometric measurements in a sample of infants with moderate positional plagiocephaly have shown excellent intra- and interrater reliability for maximal cranial length, maximal cranial width, and right and left cranial diagonals, and good intra- and interrater reliability in maximal cranial circumference measurement.

## 1. Introduction

Head and neck asymmetries are very common in healthy newborns [1]. Among these asymmetries, positional plagiocephaly (PP), a general term that describes the deformation of the skull and face, stands out, resulting from the application of prenatal or postnatal forces on the baby’s head [2]. PP is characterized by asymmetric occipital flattening, accompanied by anterior displacement of the ear on the same side, contralateral parietal protrusion, and often ipsilateral frontal protrusion, with contralateral frontal flattening. These characteristics give the head a parallelogram shape when viewed from the top [3] and it can also be seen on the face with facial asymmetry [4]. Prevalence data are limited, depend on the geographical area, and are reported with a wide range. Data reported in the literature varies from low (13–16%) [5,6,7] and median percentages (20–30%) of infants [8,9] to very high (61%) [1].

In addition to the classification of PP by visual estimation [10] which has only shown moderate reliability for clinical practice [11], there are different systems to objectify the cranial deformity: direct measurements with caliper, measurement of cranial asymmetry taking indirect references from photographs [12], 3D photographs [13], scanner images [14] or from a plastic modeling of the baby’s head shape in a system called plagiocephalometry [15,16].

Anthropometric measurements with calipers have been used frequently to assess head shape but there is controversy regarding the reliability of the data [17] and lack of homogeneity regarding the anthropometric references used [18,19,20,21,22]. Craniometry with caliper is safe, fast, and low cost, which makes it an efficient method for clinical settings.

From the data obtained with anthropometry, data are extracted for the calculation of cranial indices or ratios. The cephalic index (CI) is calculated from the equation: cranial width/cranial length × 100 [23] and determines the cranial morphology in terms of a more brachycephaly (CI > 85%) or dolichocephalic skull (CI < 75%) [23]. On the other hand, the cranial asymmetry indices or ratios require the diagonal diameters to be determined. The most used in the bibliography is the Cranial Vault Asymmetry Index (CVAI) [24,25]. The CVAI is calculated with the formula: cranial diagonal diameters difference/short diagonal diameter x 100 [23]. CVAI classifies plagiocephaly severity pursuant the Children’s Healthcare of Atlanta scale in: level 1: <3.5%; level 2: 3.5 to 6.25%; level 3: 6.25 to 8.75%; level 4: 8.75 to 11.0%; level 5: >11.0% [26]. Classification of plagiocephaly severity may guide clinicians in the decision-making process regarding the treatment options of cranial asymmetry: repositioning, physical therapy, or cranial orthosis. Level 1 is considered within normal limits and no treatment is required. Level 2 requires repositioning at least. Level 3 calls for cranial remolding orthosis depending on age and history and levels 4 and 5 need cranial remolding orthosis [26]. Even with the use of cranial remolding orthosis, repositioning and physical therapy are recommended [27]. Early intervention translates into a significant improvement in PP regardless of the severity of the asymmetries [28]. Quantifying head shape is important for clinical management of PP and direct cranial anthropometric measurements provides an efficient solution for clinical settings.

The objective of the present study was to analyze the interrater and intrarater reliability of direct anthropometric measurements with caliper on defined craniofacial references, necessary for evaluation of PP in infants and for calculation of the most common indices used for the evaluation of cranial asymmetry.

## 2. Materials and Methods

### 2.1. Subjects

A cohort of 62 subjects under 28 weeks old with signs of PP were recruited. They were consecutively referred by pediatricians from sector III of the Aragonese Health Service.

According to the method of Walter et al. (1998), developed to calculate the required number of subjects for a reliability study, where reliability is measured using the intraclass correlation coefficient, with 0.8 being the minimum acceptable level of reliability and 0.9 the maximal expected level of reliability, 45.8 subjects are needed, admitting the following values for type I and type II errors: α = 0.05 and β = 0.20 [29]. In this study, 62 subjects were recruited for the interrater and intrarater reliability study of anthropometric values, which is a sufficient sample to guarantee a good or almost perfect degree of agreement [30].

The inclusion criterion was to show a difference of at least 5 mm between cranial diagonal diameters [17]. Subjects with craniosynostosis, genetic, infectious, metabolic, or neurological diseases were excluded.

An informative document about the study was provided to the parents and an informed consent was signed after they had read the document and their questions about the study had been answered. Regulations and guidelines regarding freedom, absence of coercion, disclosure of economic interests, understandable and complete information, confidentiality and acceptance were followed [31].

The Ethics Committee at the Aragon Health Sciences Institute approved the study (Registry No. C.P.–C.I. PI16/0275).

### 2.2. Measurements

**Figure 1:**
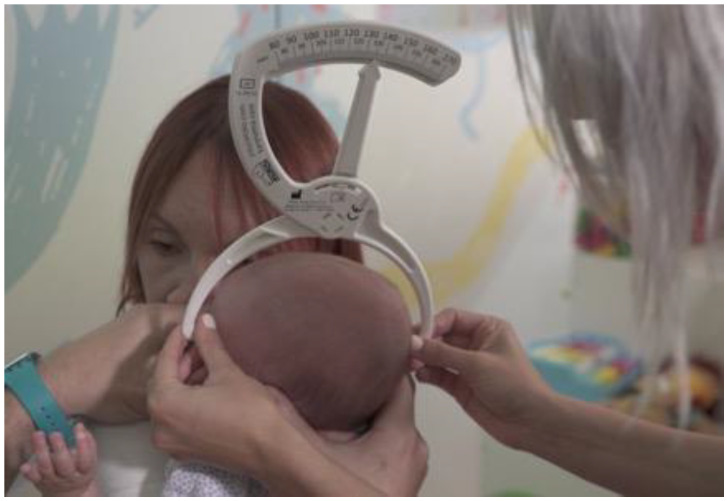
Lateral view of diagonal cranial diameter evaluation, from the frontozygomatic point ipsilateral to the contralateral lambdoid suture, on the same horizontal plane.

**Figure 2:**
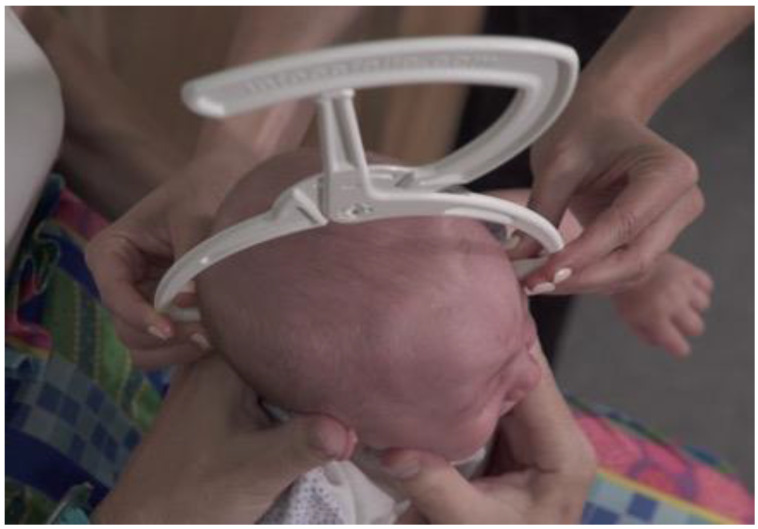
Top view of diagonal cranial diameter evaluation, from the frontozygomatic point ipsilateral to the contralateral lambdoid suture, on the same horizontal plane.

The following anthropometric measurements were taken: maximal cranial circumference (MCC) (Martini et al., 2018); maximal cranial length (MCL): linear distance between glabella and opisthocranion; maximal cranial width (MCW): linear distance between the two euryon; diagonal cranial diameters (also called cranial diagonals) taken from the frontozygomatic suture to the contralateral lambdoid suture (Figure 1 and Figure 2) [25].

From these data, the cranial vault asymmetry (CVA) (the difference between the cranial diagonal diameters) [32], the cephalic index (CI) and the CVAI were calculated. CI was calculated with the formula: cranial width/cranial length × 100 [33], while CVAI was calculated using the formula: cranial diagonal diameters difference/short diagonal diameter × 100 [23].

Measurements were made in a consultation of a clinical physiotherapy center, maintaining adequate lighting conditions for the procedure and as close as possible to the clinical daily routine. The subjects were evaluated by two trained raters in anthropometric evaluation of infants with plagiocephaly. Raters trained the measurements to agree on references search and measurement technique. They had 4 years of experience in the measurement of infants with plagiocephaly. An inextensible tape measure for the MCC and the caliper “mimos craniometer”, manufactured by Think Pipe Line SLU, were used.

For the interrater reliability study, the measurements were performed first by rater 1 (measurement 1) and later by rater 2, without exchanging information, with no time interval between both measurements. For the intrarater reliability study, rater 1 measured the same parameters again next day (measurement 2). 24 h was considered sufficient time to not remember the data taken in the first measurement and to guarantee the reliability of the comparison of measurements.

In each measurement session, MCC, MCL, MCW were taken two times and cranial diagonal diameters were taken three times, by each examiner, non-consecutively, alternating the measurements of the different parameters and the mean of the three was recorded to carry out the statistical analysis of reliability.

### 2.3. Statistical Analyses

A descriptive analysis of qualitative variables, offering the absolute frequencies and the percentages in each category and of quantitative variables, offering the mean ± standard deviation or median value (Q1–Q3) depending on whether the distribution was normal or non-normal, respectively, was carried out. Data distribution was analyzed with the Kolmogorov-Smirnov test with the Lilliefors correction, values of *p* < 0.05 were considered significant.

For intra- and interrater reliability analysis, if variables had a normal distribution, the intraclass correlation coefficient (ICC), one-factor model, random effects, was obtained. A 95% confidence interval was established for ICC. ICC has been interpreted according to the ranges established by Koo and Li [30]: values less than 0.5 are indicative of low reliability, values between 0.5 and 0.75 of moderate agreement, values between 0.75 and 0.9 of good agreement, and values greater than 0.90 of excellent reliability.

If any of the variables was not normally distributed, Bland-Altman plot was made to evaluate degree of agreement of the measurements. In Bland-Altman plots, three parallel lines were represented:

Upper limit of agreement: mean difference + 1.96 × SD.

Mean difference: mean value determined by one data series—mean value determined by the other data series. It reflects the systematic error.

Lower limit of agreement: mean difference − 1.96 × SD.

If two data series for which reliability is being studied obtain similar values on average, then the mean difference will be zero or close to zero. If it is far from this value, it would mean that the two methods produce different results.

The numerical analysis was performed using SPSS 22.0 for Windows and the Bland-Altman plots were performed with MedCalc for Windows.

## 3. Results

The interrater (two raters consecutively) and intrarater (same rater, on two consecutive days, at same time) reliability studies of the anthropometric measurements were carried out in 62 subjects.

Median age of the subjects at the time of measurement was 16 weeks (Table 1). 43.5% were female and 56.5% were male (Table 1). Descriptive values of cranial asymmetry indices can be consulted in Table 1.

Descriptive values for the two MCC measurements of rater 1 are shown in Table 2.

**Figure 3:**
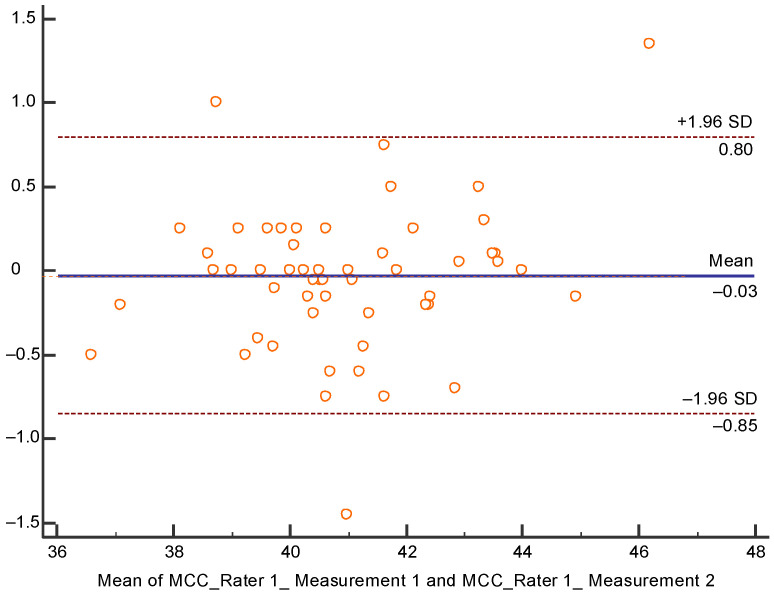
Bland-Altman plot of MCC intrarater reliability. SD: Standard deviation.

Reliability was analyzed with the Bland-Altman Plot (Figure 3), since measurement 1 of rater 1 did not show a normal distribution, which would have allowed ICC calculation. However, the plot shows a good degree of agreement since the mean of the differences is very close to 0, being −0.03 cm.

The intrarater reliability for the rest of the variables was analyzed with the ICC since all variables followed a normal distribution. Confidence intervals of the ICC were calculated, as well as its *p* values (Table 3).

Descriptive values of MCC and left cranial diagonal measurements of rater 1 and rater 2 are shown in Table 4.

**Figure 4:**
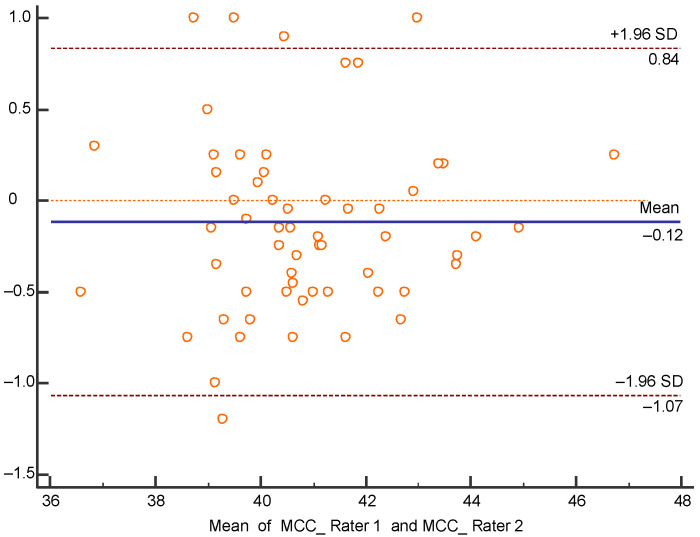
Bland-Altman plot of MCC interrater reliability.

**Figure 5:**
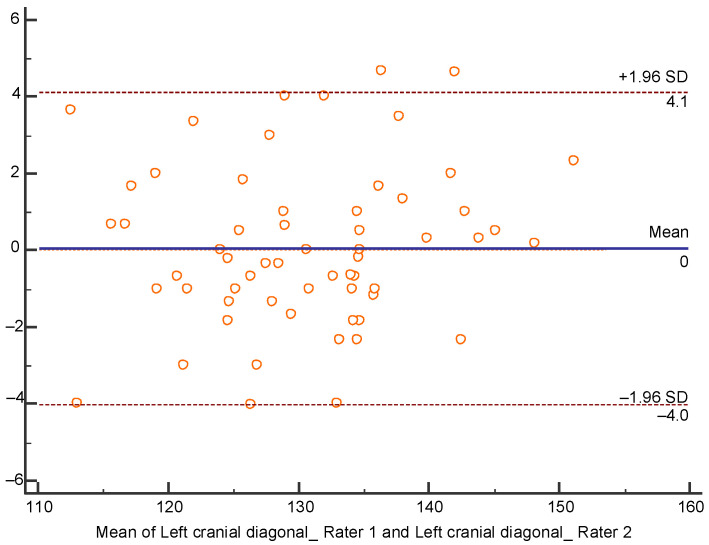
Bland-Altman plot of left cranial diagonal interrater reliability.

Reliability was analyzed with Bland-Altman Plots (Figure 4 and Figure 5), because MCC measurement of rater 1 and left cranial diagonal measurement of rater 2 did not show a normal distribution, which would have allowed ICC calculation. Plot referring to MCC (Figure 4) shows a good degree of agreement since the mean of the differences is very close to 0, being −0.12 cm.

Plot referring to left cranial diagonal (Figure 5) shows an excellent degree of agreement, since mean difference is 0.0 cm.

The interrater reliability for the rest of the variables was analyzed with the ICC since all the variables followed a normal distribution. Confidence intervals of ICCs were calculated, as well as its *p* values (Table 5).

## 4. Discussion

The sample of this study consisted of 62 children with PP, with a difference of at least 5 mm between diagonal cranial diameters, i.e., children with at least moderate deformity [17]. 43.5% were female and 56.5% were male, so the slightly higher prevalence in male is according to data reported in the literature [34]. 75% of the sample was older than 13 weeks, so most of the sample had already exceeded three months, age below which PP could be found in almost half of infants [35].

Mean CI was 86.8%. Normal range described is between 75 and 85% [25], therefore, infants in the study had a tendency toward brachycephaly: they had the skull with a predominance of width over length.

Median CVA was 8.19 mm. According to Mortenson and Steinbok, who classify CVA into the following categories: normal CVA < 3 mm, mild/moderate CVA ≥ 3 mm and CVA ≤ 12 mm, moderate/severe CVA > 12 mm [17], the sample had a moderate PP.

Median CVAI was 6.43%. Plagiocephaly severity scale, according to CVAI is: level 1: <3.5%; level 2: 3.5 to 6.25%; level 3: 6.25 to 8.75%; level 4: 8.75 to 11.0%; level 5: >11.0% [26]. Therefore, infants in this study presented a level 3, which depending on the age and medical history, may require cranial remodeling orthoses for their treatment.

Intrarater reliability was excellent for MCL and MCW, and for left and right cranial diagonal diameters. Good reliability was observed in MCC measurement.

Interrater reliability was excellent for MCL and MCW, and for left and right cranial diagonal diameters. Good reliability was observed in MCC measurement.

Slightly worse records in the reliability of the MCM were due to technical difficulty in the measurement. Exact references are not used for its recording. Maximal value of the fronto-occipital circumference is sought, which easily results in greater variability.

Our data confirm good results obtained by Mortenson and Steinbok (2006) regarding intrarater reliability and are superior to them regarding CVA interrater reliability, in infants referred for plagiocephaly or torticollis [17]. These authors took the distance between the frontozygomatic point (most medial point of the temporal crest of the frontal bone) and contralateral euryon (most lateral point of the neurocranium, it can be located in the parietal or in the temporal squama) as anthropometric references to establish the diagonals [17], while in this study the distance between the frontozygomatic point and the inner rim of the lambdoid suture of the contralateral side has been used, according to Wilbrand et al. [25], who reported good intra- and interrater reliability for measurements of circumference, length, width, and diagonal distances.

Skolnick proposed the distance between the contralateral frontozygomatic-euryon points as the best correlated with cranial perimeter [22,36], while in this study references proposed by Wilbrand were chosen, since the distance between the frontozygomatic-euryon points does not seem to show the global characteristic of the cranial deformation in its posterior part [22,36].

Skolnick et al. (2015) conducted a comparison study between direct anthropometric measurements and digital measurements. The study included caliper measurements of the length, width and diagonals and the measurement of the circumference by meter. In the results they found an excellent reproducibility of all the caliper measurements, and they appreciated a strong correlation between direct and digital measurements (R2 > 0.90). Caliper measurements were 1 to 4 mm shorter than digital with consistent variation [37]. Mendonca et al. (2013) in a previous study found less correlation between direct and digital measurements, with a significant difference of 6% in measurements of anteroposterior length and cranial width [38].

Direct anthropometric measurements on the skull using a caliper are a reliable tool for diagnosis and decision-making in plagiocephaly [25]. Results of our study with an analysis in a larger sample of children with PP contrast these previous results. It is an easy, effective, low cost and reproducible method if the examiners and assistants are well trained. These advantages favor that this measurement system seems to be the most used by the American Society of Maxillofacial Surgeons [39].

## 5. Conclusions

Anthropometric measurements taken in a sample of infants with moderate severity PP have shown excellent intra- and interrater reliability for MCL, MCW, and right and left cranial diagonals, and good intra- and interrater reliability in MCC measurement.

## Figures and Tables

**Table 1:** Sample description.

Infants with PP (*n* = 62)		Descriptive
Gender	Females (*n* = 27)	43.5%
Males (*n* = 35)		56.5%
Age (weeks)		16.0 (13.0; 19.0) **
CI (%)		86.8 ± 7.4 *
CVA (mm)		8.2 (6.0; 11.6) **
CVAI (%)		6.4 (5.0; 9.4) **

* Mean ± standard deviation. ** Median (Q1; Q3). CI: Cephalic index; CVA: Cranial vault asymmetry; CVAI: Cranial vault asymmetry index; PP: Positional plagiocephaly.

**Table 2:** Data for the intrarater reliability study of MCC.

Infants with PP (*n* = 62)	Rater 1	Descriptive	Kolmogorov-Smirnov. Sig.
MCC	Measurement 1	40.4 (39.4; 42.0) **	0.016
	Measurement 2	40.8 ± 1.8 *	0.200

* Mean ± standard deviation. ** Median (Q1; Q3). MCC: Maximal cranial circumference.

**Table 3:** Intrarater reliability study of MCL, MCW, right cranial diagonal and left cranial diagonal.

Infants with PP (*n* = 62)	Rater 1	Descriptive	Kolmogorov-Smirnov. Sig.	ICC	95% Confidence Interval		*p* Value
					Lower Limit	Upper Limit	
MCL	Measurement 1	132.6 ± 7.5 *	0.077	0.987	0.978	0.992	0.000
	Measurement 2	132.8 ± 7.4 *	0.200				
MCW	Measurement 1	114.8 ± 8.1 *	0.200	0.977	0.962	0.986	0.000
	Measurement 2	115.5 ± 7.6 *	0.200				
Right cranial diagonal	Measurement 1	127.6 ± 8.2 *	0.200	0.990	0.983	0.994	0.000
	Measurement 2	128.1 ± 7.9 *	0.200				
Left cranial diagonal	Measurement 1	130.5 ± 8.7 *	0.200	0.983	0.972	0.990	0.000
	Measurement 2	131.1 ± 8.2 *	0.200				

* Mean ± standard deviation. ICC: Intraclass correlation coefficient; MCL: Maximal cranial length; MCW: Maximal cranial width.

**Table 4:** Data for the interrater reliability study of MCC and left cranial diagonal.

Infants with PP (*n* = 62)	Raters	Descriptive	Kolmogorov-Smirnov. Sig.
MCC	Rater 1	40.4 (39.4; 42.0) **	0.016
	Rater 2	40.9 ± 1.8 *	0.100
Left cranial diagonal	Rater 1	130.5 ± 8.7 *	0.200
	Rater 2	130.5 (125.1; 135.4) **	0.038

* Mean ± standard deviation. ** Median (Q1; Q3).

**Table 5:** Interrater reliability study of maximal cranial length, maximal cranial width, right cranial diagonal.

Infants with PP (*n* = 62)	Raters	Descriptive	Kolmogorov-Smirnov. Sig.	ICC	95% Confidence Interval		*p* Value
					Lower Limit	Upper Limit	
MCL	Rater 1	132.6 ± 7.5 *	0.077	0.983	0.972	0.990	0.000
	Rater 2	133.4 ± 7.0 *	0.200				
MCW	Rater 1	114.8 ± 8.1 *	0.200	0.978	0.963	0.987	0.000
	Rater 2	116.2 ± 7.8 *	0.200				
Right cranial diagonal	Rater 1	127.6 ± 8.2 *	0.200	0.986	0.977	0.992	0.000
	Rater 2	127.4 ± 8.2 *	0.200				

* Mean ± standard deviation.
